# On Identifying the Optimal Number of Population Clusters via the Deviance Information Criterion

**DOI:** 10.1371/journal.pone.0021014

**Published:** 2011-06-28

**Authors:** Hong Gao, Katarzyna Bryc, Carlos D. Bustamante

**Affiliations:** 1 Stanford Genome Technology Center and Department of Biochemistry, Stanford University, Stanford, California, United States of America; 2 Department of Genetics, Harvard Medical School, Boston, Massachusetts, United States of America; 3 Department of Genetics, Stanford University, Stanford, California, United States of America; University of Utah, United States of America

## Abstract

Inferring population structure using Bayesian clustering programs often requires *a priori* specification of the number of subpopulations, 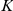, from which the sample has been drawn. Here, we explore the utility of a common Bayesian model selection criterion, the Deviance Information Criterion (DIC), for estimating 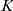. We evaluate the accuracy of DIC, as well as other popular approaches, on datasets generated by coalescent simulations under various demographic scenarios. We find that DIC outperforms competing methods in many genetic contexts, validating its application in assessing population structure.

## Introduction

A common problem in modern population genetics is identifying population substructure among a sample of individuals genotyped across a set of neutral genetic markers. Bayesian clustering algorithms such as STRUCTURE [1], [2] and BAPS [3] and their derivates [4]–[8] are commonly used for addressing this problem. Of particular concern to many investigators is estimating the number of subpopulations or clusters 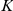 that are necessary and sufficient to explain observed patterns of genetic variation. Part of the reason investigators are concerned with the “choosing 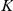
” problem is that many of the classification algorithms (including STRUCTURE) require specifying the number of clusters as a parameter in the model. A consequence of this is that the biological conclusions one draws from the data may be artificially dependent on the value of 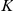 chosen. In practice, many investigators analyze their data using a range of values for 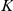, reporting the output for all (or a plausible set of) 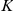
's and/or employ one of several *post hoc* statistics [1], [4], [9] to choose an optimal value for 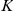. The purpose of this communication is to report our experience with the Deviance Information Criterion (DIC) as a statistic for choosing 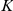. By comparing the performance of DIC to other commonly used statistics on simulated data under a variety of population genetic scenarios, we find that it often outperforms other approaches and recommend it be considered by investigators interested in estimating 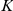 from genotype data. Its advantage over more complex approaches such as the reversible-jump Markov chain Monte Carlo (MCMC) or the Dirichlet process prior on 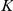, is that calculating DIC requires trivial computational overhead once the MCMC has been run.

Choosing 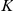 is a difficult problem in the Bayesian clustering setting, because as 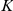 increases, the likelihood of the data increases monotonically, as well as the complexity of the model. Adding more degrees of freedom to the analysis generally improves the overall fit of the model to data. This often results in monotonic non-decrease in the probability of the data given 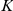 as 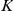 increases [1], [9]. A common way of dealing with this class of statistical problems (known as “model selection”) is to use a penalizing function which weighs the fit of a model versus its complexity. This is the underlying idea behind many model selection statistics such as the Akaike Information Criterion (AIC) and the Bayesian Information Criterion (BIC). The Deviance Information Criterion (DIC) is a recently proposed statistic for model selection when the posterior distribution of parameters in competing models are estimated using Markov chain Monte Carlo, as is the case with STRUCTURE and its derivatives [10].

## Results

We applied the Deviance Information Criterion to estimate 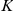 for datasets generated by coalescent simulations under various demographic scenarios and for the large-scale genotype data from Human Genome Diversity Panel. We evaluated the accuracy of DIC in comparison with other popular approaches and demonstrate that DIC performs well in a variety of scenarios.

### Application to Simulated Data

**Figure 1:**
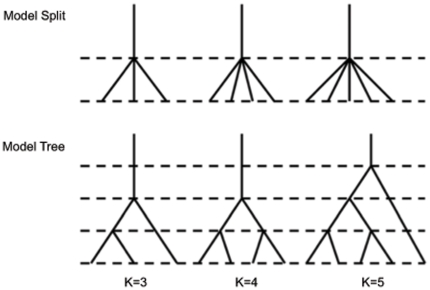
Subpopulation topology of Model Split and Model Tree for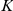 ranging from three to five. In Model Split, subpopulations are split from one ancestral population simultaneously, forming a star-shaped topology. In Model Tree, populations separate at different time points, forming a tree-shaped topology. The time interval between two consecutive dashed lines is 0.5 scaled in units of 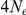 generations, where 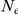 is the effective population size.

**Figure 2:**
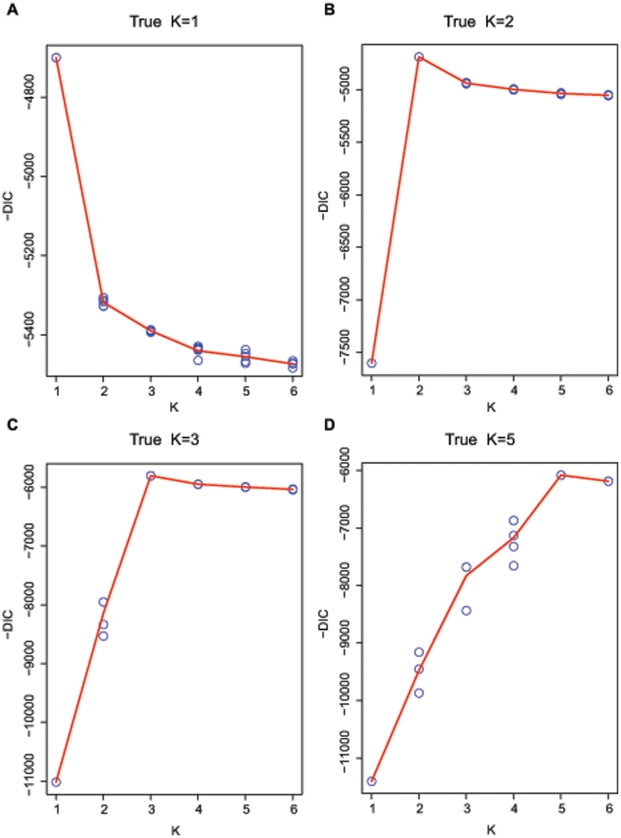
Performance of DIC on one data set simulated under Model Split for each true 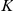 value, 1,2,3 and 5.

We performed extensive coalescent simulations using multiple demographic models, including Models Split, Tree, 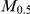, 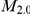, 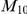 and Inbred (see Section Methods and Figure 1). Models Split and Tree implement the distinct demographic histories during subpopulation formation. Models 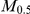, 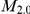, and 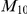 are used to investigate the impact of different levels of exchange among subpopulations on the inference of population structure. Model Inbred is designed to test the effect of the confounding factor “inbreeding”. To evaluate the robustness of our method in the case of scarcity of data, we also simulate the Model Split with 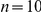 individuals or 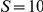 SNPs. The last scenario tested is to reduce the splitting time among subpopulations by a factor of ten. This is equivalent to decreasing the genetic distances among subpopulations, which implicitly reflects the various levels of physical distances among populations. Then we ran each data set through InStruct [5] with five MCMC chains for each value of 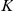, retaining a total of 50,000 iterations after a 500,000 iteration burn-in period with a thinning interval of ten iterations between retained draws. Figure 2 illustrates the performance of DIC on a randomly selected data set generated under Model Split with true 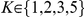. For these four data sets, 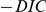 always peaked at the correct 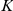 values for all the chains. (Note that we choose to plot 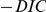 because it is often easier to visualize a maximum peak than a minimum trough).

To place our work in a broader context, we also ran these data sets through five methods commonly used to estimate 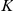: (1) the approximate likelihood method implemented in STRUCTURE using both the original and correlated allele frequency model, (i.e., the “F” model [1], [2]), (2) the 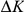 approach based on running STRUCTURE with both the original and F models [9], (3) Eigenanalysis method (implemented in “SmartPCA” software) proposed by Patterson et al. [11] which estimates 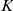 as 1 plus the number of significant eigenvalues underlying a principal component decomposition (PCD) of the scaled genotypic value matrix, (4) Structurama which uses a Dirichlet process prior model to partition a sample into subgroups [12], [13], and (5) BAPS utilizing the splitting and merging strategy to attain the best classification [3], [6]–[8]. We also conducted preliminary analyses using the regularization method [4], but found that it consistently performed poorly for moderate values of 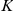 (e.g., the accuracy was under 50% when 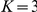 under the Split model).

We assessed the accuracy of each method as the proportion of data sets which correctly recover the value of 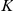 used in data simulation using the optimality criterion defined for each approach. For example, for DIC, we used the lowest DIC value observed across five independent MCMC chains run for each of the six values of 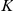. For Eigenanalysis, we assessed accuracy under three significance levels (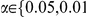 and 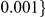). For Structurama, we chose the partitions of individuals with the highest posterior probabilities under two prior distributions: (1) a noninformative prior on the number of clusters, and (2) a prior distribution with the expected number of clusters equal to the true value of 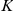 used to simulate the data. We use the individual clustering mode of BAPS as our simulation does not include admixture.

Under the case of simple population splitting with a high degree of population differentiation, i.e., 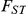 values around 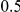, we found that the DIC method consistently outperformed other approaches (see Table 1). For example, under Model Split, the accuracy is near 100% for all values of 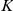 considered. STRUCTURE, on the other hand, has an accuracy that ranges from 54% to 100% depending on the true 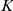 and whether or not the 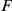 model is employed. We also observe that the accuracy of 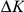 decays with 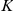, starting at 100% for 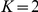 and reaching 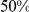 and 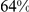 for the 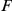 and non-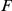 models, respectively, at 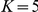. Eigenanalysis tends to perform well, but is sensitive to the choice of 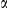 with smaller values (e.g., 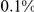) of 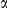 performing better than higher values (e.g., 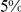). The performance of Structurama on simulated data was interesting. It performed perfectly well when 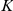 was small (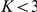) but when 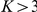, it tended to fail almost completely. We posit that this may be due to the tendency of the Dirichlet process mixture model to overcluster, which results in 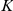 being underestimated. An alternative explanation is that the Dirichlet process prior fails to converge within a finite number of iterations in practice, which commonly challenges many other mixture model methods [14]. BAPS performs perfectly well, except in the case of 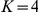, it drops to 82%. The performance of most methods under the complex splitting model (i.e., Model Tree) was similar to the performance under Model Split. This implies our results are robust to moderate deviations from the 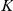-wise subpopulation split topology assumed in STRUCTURE.

**Table 1:** Accuracy of multiple 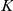 estimators under Models Split and Tree.

Model	Split					Tree		
K	1	2	3	4	5	3	4	5
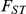		0.495	0.502	0.493	0.492	0.486	0.507	0.501
DIC	1.00	1.00	1.00	1.00	1.00	1.00	1.00	0.98
STRUCTURE	0.90	1.00	1.00	0.86	0.80	0.98	0.94	0.72
STRUCTURE, F model	0.90	0.98	0.94	0.82	0.54	0.90	0.82	0.62
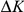		1.00	0.94	0.70	0.64	0.80	0.86	0.64
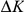 , F model		1.00	0.90	0.78	0.50	0.84	0.92	0.54
Eigenanalysis, 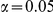	0.97	0.89	0.86	0.86	0.96	0.96	0.92	0.90
Eigenanalysis, 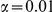	1.00	0.96	0.91	0.93	0.99	0.98	0.94	0.92
Eigenanalysis, 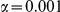	1.00	1.00	0.96	0.96	1.00	1.00	0.96	0.96
Structurama, noninformative prior	1.00	1.00	0.82	0.18	0.02	0.88	0.22	0.00
Structurama, correct prior	1.00	1.00	0.82	0.18	0.02	0.82	0.22	0.00
BAPS	1.00	1.00	1.00	0.82	1.00	1.00	1.00	0.96

Performance assessment of methods including DIC, STRUCTURE, 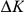, Eigenanalysis, Structurama and BAPS. “
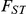
” is the population differentiation statistic estimated by SmartPCA [11] averaged across 50 data sets. STRUCTURE's performance is evaluated based upon both the original model and the correlated alleles or “F” model. Similarly tested is the 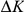 statistic that relies on STRUCTURE. Eigenanalysis is tested at three significance levels (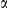). Structurama is assessed using both a noninformative prior on 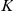 and the true 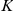 value as the starting point. BAPS is evaluated using the individual clustering mode. Blank values in the table indicate that a program did not generate a result.

Migration among subpopulations, on the other hand, can have a profound impact on the accuracy of all approaches. When migration rates are low between subpopulations (Model 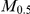), DIC, BAPS, and Eigenanalysis with a stringent p-value cutoff both worked perfectly. STRUCTURE also performed reasonably well with accuracy rates ranging between 84% and 100% (see Table 2). When the migration rates among subpopulations are intermediate (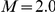 corresponding to 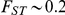), most methods showed results similar to those under Model 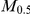. The notable exception was Structurama which performed poorly (at least under the parameter values we explored). Under low population differentiation (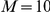; 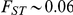), all methods showed a decrease in accuracy. For example, the accuracy of DIC noticeably decreases with 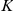 reaching a low of 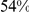 for 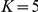 (see Table 3). The original STRUCTURE model also performed poorly with accuracy well below 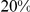. Interestingly, in the case of strong migration, the 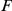 model's accuracy is much higher both for the STRUCTURE and 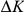 statistics. This is probably because the correlated alleles model is doing a good job in modeling patterns of genetic variation among admixed subpopulations. Since InStruct does not implement an 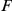 model, we predict that adding the 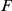 model to InStruct or implementing DIC within STRUCTURE with the 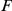 model would perform as well or better than these statistics. Eigenanalysis also seems to handle the high migration rate scenario well. Its accuracy decreases only slightly with 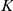, compared to the low migration rate case. Intriguingly, the most stringent significance level for high migration does not necessarily perform best, as it does with the slower migration models. This suggests that it may be challenging to find the optimal tuning of 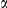 for best classification accuracy when using PCD and a Tracy-Widom approximation to the distribution of p-values. We also observe that Structurama appears to be very sensitive to migration. It clusters all individuals into one group for every data set under Model 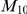, i.e. no matter which prior is used, Structurama incorrectly estimates 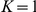 for every simulated data set. Our results differ from [12], who found Structurama worked well in estimating 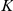 under certain scenarios. We believe the differences may be due to the details of the simulation used. They considered an island model with migration, whereas we used a population-split model with subsequent migration among demes. This slight difference leads to more subpopulation differentiation in their simulations than ours, since they have a longer expected coalescent time between demes than we do. (That is, in our simulations all demes merge, looking back in time, at the time of population splitting). BAPS's accuracy decreases sharply as 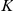 increases, implying that it performs poorly in the case of weak population differentiation.

**Table 2:** Accuracy of multiple 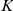 estimators under Models 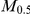 and 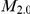.

Model	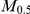 , slow migration				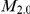 , moderate migration			
K	2	3	4	5	2	3	4	5
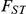	0.392	0.430	0.452	0.454	0.191	0.248	0.263	0.281
DIC	1.00	1.00	1.00	1.00	1.00	1.00	1.00	1.00
STRUCTURE	1.00	0.98	0.94	0.84	1.00	1.00	0.96	0.84
STRUCTURE, F model	0.88	0.96	0.94	0.88	0.86	0.86	0.94	0.86
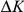	1.00	0.78	0.94	0.80	1.00	0.92	0.76	0.80
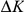 , F model	1.00	0.84	0.94	0.88	1.00	0.96	0.80	0.92
Eigenanalysis, 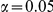	0.96	0.84	0.98	0.96	1.00	0.86	0.94	0.98
Eigenanalysis, 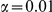	0.98	0.94	1.00	0.96	1.00	0.98	0.98	1.00
Eigenanalysis, 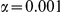	0.98	1.00	1.00	1.00	1.00	1.00	1.00	1.00
Structurama, noninformative prior	1.00	0.96	0.80	0.44	0.74	0.52	0.12	0.00
Structurama, correct prior	1.00	0.98	0.78	0.44	0.72	0.52	0.10	0.06
BAPS	1.00	1.00	1.00	1.00	1.00	1.00	1.00	0.98

Evaluation of these methods are performed in the same manner as in Table 1.

**Table 3:** Accuracy of multiple 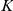 estimators under Models 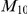 and Inbred.

Model	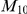 , fast migration				Inbred				
K	2	3	4	5	1	2	3	4	5
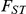	0.048	0.063	0.069	0.073		0.489	0.498	0.491	0.504
DIC	1.00	0.94	0.70	0.56	1.00	1.00	1.00	0.98	0.98
STRUCTURE	0.02	0.02	0.06	0.16	0.64	1.00	0.98	0.90	0.84
STRUCTURE, F model	0.90	0.98	1.00	1.00	0.34	0.36	0.22	0.20	0.22
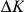	0.32	0.48	0.26	0.16		1.00	0.74	0.80	0.68
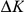 , F model	0.94	0.96	0.74	0.64		1.00	0.94	0.84	0.82
Eigenanalysis, 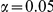	0.94	0.96	0.90	0.94	0.86	0.68	0.61	0.66	0.68
Eigenanalysis, 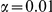	1.00	0.98	0.90	0.90	0.96	0.92	0.73	0.78	0.75
Eigenanalysis, 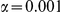	1.00	0.92	0.90	0.84	1.00	0.93	0.81	0.84	0.85
Structurama, noninformative prior	0.00	0.00	0.00	0.00	1.00	1.00	0.82	0.24	0.02
Structurama, correct prior	0.00	0.00	0.00	0.00	1.00	1.00	0.78	0.22	0.02
BAPS	0.64	0.54	0.22	0.14	0.74	1.00	1.00	1.00	0.98

Evaluation of these methods are performed in the same manner as in Table 1.

When we assessed accuracy under the inbreeding model, assuming undetected inbreeding (such as partial self-fertilization) within subpopulations, we found again that DIC tends to outperform other methods (see Table 3). It is important to note that in calculating DIC, we have used InStruct's inbreeding model whereas the other approaches based on STRUCTURE assume the Hardy-Weinberg equilibrium within clusters. We, and others, have shown that failing to consider inbreeding in the likelihood calculation for STRUCTURE can lead to spurious signals of population admixture and erroneous inference of the number of subpopulations [5]. This phenomenon appears to cause a large reduction in the accuracy of estimating 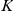 by STRUCTURE's 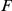 model with only 20% of simulations uncovering the true number of populations underlying the data. Eigenanalysis, which does not account for inbreeding either, likewise overestimates the number of subpopulations, and has an accuracy ranging from 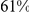 to 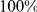 depending on the true value of 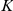. Both Structurama and BAPS are not heavily affected by hidden inbreeding, and have the similar accuracy pattern as under Model Split.

To assess the robustness of DIC in the limit of small data sets, we simulated data under Model Split for 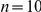 individuals or 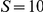 SNPs. We found that the accuracy of DIC is robust to the former, but not the latter (Table 4). When the subpopulation size decreases to 10, DIC performs almost as well as with a larger number of individuals per subpopulation. STRUCTURE and 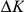, on the other hand, show a significant reduction in accuracy as 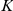 increases to 5. Eigenanalysis shows a reduction in accuracy only when using a stringent p-value cutoff. When the number of markers is reduced to only 10, DIC's accuracy falls to 42% when 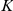 increases to five, which is expected as DIC is an asymptotic approximation that only holds as the sample size is sufficiently large, and the accuracy of STRUCTURE and 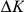 is close to zero. With so few markers, Eigenanalysis fails to provide an output. Structurama also performs poorly under larger values of 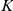
s. BAPS is robust to the decrease in sample size but is strongly affected by reducing the number of markers. While we conclude that DIC is more robust than other approaches to small data sizes, we, of course, expect accuracy to increase with 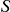 and so recommend that investigators genotype as many unlinked markers as is economically feasible.

**Table 4:** Accuracy of multiple 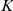 estimators with reduced data dimensions.

Model	Subpopulation Size = 10					Number of Loci = 10				
K	1	2	3	4	5	1	2	3	4	5
DIC	1.00	1.00	1.00	1.00	0.98	1.00	1.00	0.82	0.42	0.48
STRUCTURE	0.84	1.00	0.86	0.60	0.40	1.00	0.96	0.86	0.72	0.18
STRUCTURE, F model	0.16	1.00	0.86	0.66	0.34	0.10	0.96	0.86	0.72	0.18
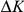		0.98	0.68	0.64	0.22		0.94	0.24	0.06	0.04
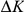 , F model		0.98	0.86	0.62	0.16		0.94	0.20	0.10	0.04
Eigenanalysis, 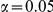		0.90	0.80	0.82	0.80					
Eigenanalysis, 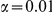		0.96	0.84	0.88	0.92					
Eigenanalysis, 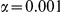		0.20	0.42	0.66	0.78					
Structurama, noninformative prior	1.00	1.00	0.96	0.38	0.00	1.00	0.90	0.40	0.14	0.00
Structurama, correct prior	1.00	1.00	0.96	0.38	0.00	1.00	0.90	0.38	0.12	0.00
BAPS	1.00	1.00	1.00	0.8	0.5	0.00	0.02	0.04	0.36	0.28

Evaluation of these methods are performed in the same manner as in Table 1. Data are simulated under Model Split with the size of each subpopulation reduced from 50 to 10 and the number of loci reduced from 100 to 10, respectively.

Under the Split model, our simulated data sets had a high degree of population differentiation (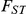 among clusters was around 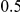). To investigate the effect of weaker population structure on estimation accuracy, we simulated data with a reduced splitting time of 0.05 in units of 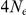 generations. This gives simulated data with 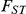 among subpopulations in the range of 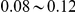. We found that shortening the splitting time, not surprisingly, reduced the accuracy of all methods with results similar to those observed for the strong migration among subpopulations (Model 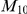). We note, in particular, that the Bayesian methods showed a decrease in accuracy with increasing 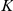. Interestingly, Eigenanalysis performed quite well, particularly using the less stringent significance level (see Table 5), which is consistent with the original results of [11] that their approach can detect very fine-scale population structure.

**Table 5:** Accuracy of multiple 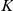 estimators with shorter splitting time among subpopulations.

Model	Subpopulation Splitting Time = 0.05				
K	1	2	3	4	5
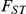		0.090	0.084	0.093	0.097
DIC	1.00	1.00	0.92	0.60	0.26
STRUCTURE	0.64	0.78	0.50	0.54	0.22
STRUCTURE, F model	0.76	1.00	0.94	0.94	0.74
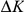		1.00	0.44	0.08	0.04
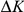 , F model		0.96	0.78	0.56	0.42
Eigenanalysis, 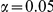	0.96	0.96	0.94	0.9	0.72
Eigenanalysis, 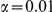	1.00	1.00	0.98	0.94	0.70
Eigenanalysis, 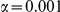	1.00	1.00	0.98	0.88	0.48
Structurama, noninformative prior	1.00	0.00	0.00	0.00	0.00
Structurama, correct prior	1.00	0.00	0.00	0.00	0.00
BAPS	1.00	1.00	0.58	0.02	0.00

Evaluation of these methods are performed in the same manner as in Table 1. Data are simulated under Model Split with the splitting time reduced from 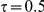 to 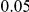.

### Application to Human Data

**Figure 3:**
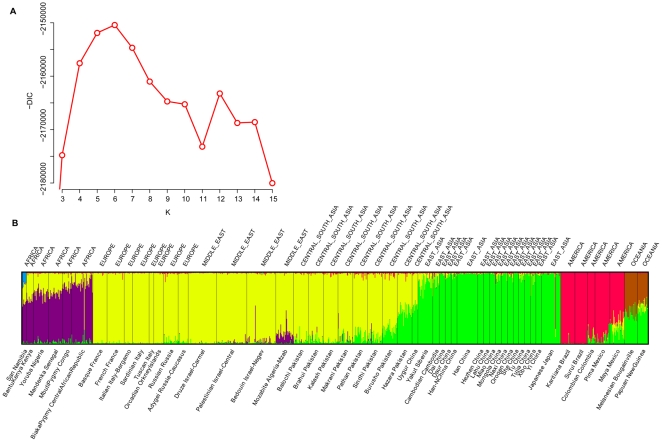
Analysis result of data from the Human Genome Diversity Panel. A. Estimated DIC for different values of 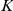. B. Distruct classification bar plot of individuals from the above data set assuming 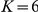. Each vertical bar represents one individual and each color represents a different cluster.

To demonstrate a concrete application of DIC, we have applied the approach with the inbreeding model of InStruct to the Human Genome Diversity Panel (HGDP-CEPH) data from [15], containing 1056 individuals from 52 populations, genotyped at 377 autosomal microsatellite loci. We find that DIC estimates 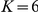 for these data as shown in Figure 3A. The five clusters we estimate (see Figure 3B) correspond approximately to the geographic regions of Africa, Europe/the Middle East/Central-South Asia, the Americas, East Asia, and Oceania as described by [15]. It is interesting to note that in our classification, we also found evidence that some alleles from the San people of Namibia, Africa, may form a sixth minor cluster with a posterior inbreeding coefficient estimate around 0.20, the highest of all clusters.

## Discussion

The Deviance Information Criterion is a simple and effective model selection method for estimating 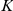, the number of clusters underlying a sample of individuals. We anticipate this approach will have wide applications in population structure inference. One important factor affecting our estimation of the accuracy of DIC is the underlying probabilistic model used in InStruct. Since InStruct takes inbreeding into account, it naturally outperforms approaches that do not model non-random mating explicitly. At the same time, since we do not implement the 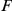 model, we do poorly when migration rates are high and allele frequencies are similar among clusters. Furthermore, the accuracy of DIC sometimes fluctuates with the quality of the classification of individuals into clusters. As in any complex MCMC framework, the likelihood surface may be multimodal for a given value of 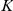. In practice, we have observed that DIC values may vary substantially among independent MCMC chains for the same dataset, especially for larger 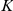 values, due to poor mixing of MCMCs under some scenarios. We recommend that for a given value of 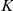, several chains be run and the minimum value of DIC across chains be used for inference. It is also important to note that population structure is a complex concept with a hierarchical form and multiple levels. DIC infers the best partition of a group of individual genetic materials taken as a whole. To investigate the finer scale of subpopulation structure, we suggest further structure analysis within each inferred cluster.

## Methods

### DIC Statistic

Here we introduce the Deviance Information Criterion formula in details. Denote 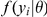 for 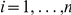 as the probability of observing individual 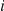
's genotype given parameters 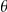 of the model which include factors such as subpopulation allele frequencies, probabilities of assignment, inbreeding coefficients, etc. For a given multivariate parameter vector 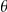, we define the deviance as:
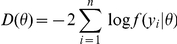
The above formula is easily recognized as the usual log-likelihood function evaluated at 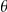. [10] defines the Deviance Information Criterion as
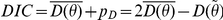
where 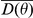 is the posterior mean deviance and 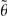 is a point estimate of the parameters. The quantity 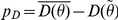 is an estimate of the “effective number of parameters in the model”. We estimate 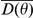 using 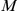 retained Markov chain Monte Carlo draws:
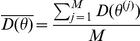
where 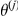 represent the retained values of the parameters at iteration 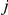. In the Bayesian clustering problem, point estimates of 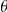 can often be ill-behaved due to the label-switching problem, and according to [16], a more stable estimator of DIC for mixture models is based on averaging the likelihood over retained draws:
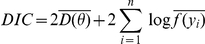
where
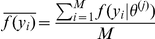
is the average value of the likelihood function for individual 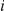 across retained draws from an MCMC chain. As with AIC and BIC, a smaller value of DIC indicates a better fitting model. We implemented the Deviance Information Criterion in our program InStruct [5] accessible through the web interface http://cbsuapps.tc.cornell.edu/InStruct.aspx.

### Data Simulation

To demonstrate the performance of DIC and compare it with other methods, we used the standard coalescent simulation program “ms” [17] to generate data under various genetic scenarios. For each population substructure scenario, we assumed a sample of 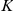 subpopulations for 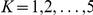, and equal and constant subpopulation sizes of 50 individuals genotyped at 100 unlinked neutral diallelic (i.e., SNP) loci. Six major genetic contexts considered in our simulation are listed below:

**Model** Split 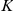 subpopulations that split without subsequent migration.

Model Tree 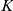 subpopulations with a tree-shaped relationship describing the splitting process.

**Model** *M_0.5_* 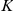 subpopulations with a scaled migration rate 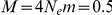 between any of two subpopulations.

**Model** *M_2.0_* 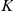 subpopulations with a scaled migration rate 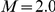 between any of two subpopulations.

**Model** *M_10_* 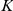 subpopulations with a scaled migration rate 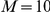 between any of two subpopulations.

**Model** Inbred 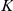 subpopulations without migration, each subpopulation with a randomly sampled selfing rate.

For Model Split, 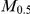, 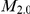, 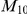 and Inbred, all subpopulations split from a common ancestral population at a time 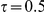 in the past scaled in units of 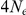 generations, where 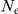 is the effective subpopulation size. In Model Inbred, partial self-fertilization within subpopulations is taken into account using the same simulation scheme as in [5]. For Models 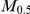, 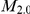 and 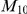, 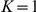 is omitted as there is no migration in the case of only one subpopulation. Besides the star-shaped genealogy among subpopulations in Model Split, Inbred, 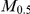, 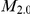 and 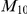, we also considered the tree topology relationship among subpopulations described in Model Tree as illustrated in Figure 1. For this model, the 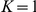 and 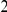 cases are ignored since they are identical to the corresponding 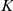
s under Model Split.

To assess the robustness of our conclusions to changes in sample size, the number of loci genotyped, or population divergence time, we undertook further simulations using Model Split. First, we reduced subpopulation size from 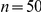 to 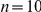. Second, we reduced the number of markers used in the analysis from 100 to 10. Third, we reduced the splitting time from the common ancestral population from 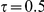 to 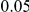. For each of the nine contexts described above (6 models+3 robustness conditions), we simulated 50 replicate data sets per value of 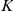.
